# Supplementary figures and images for: Polar localization of CheO under hypoxia promotes Campylobacter jejuni chemotactic behavior within host
Source: PLoS Pathog. 2022 Nov 3;18(11):e1010953. doi: 10.1371/journal.ppat.1010953 (PMC9665402; doi:10.1371/journal.ppat.1010953)

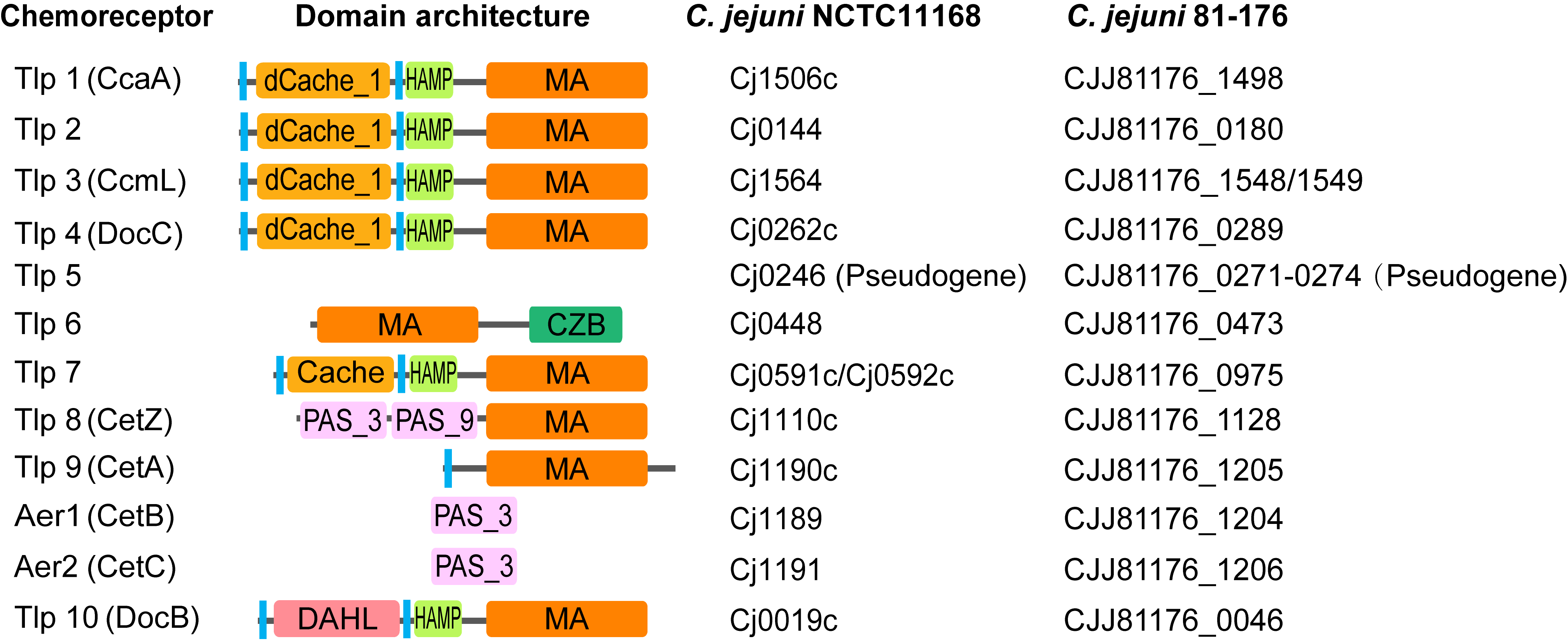

Supplement: S1 Fig — Tlp3 in C. jejuni 81–176 is annotated as two genes (CJJ81176_1548 and CJJ81176_1549), the same for Tlp7 in NCTC11168 (Cj0591c/Cj0592c). (TIF) [file ppat.1010953.s001.tif]

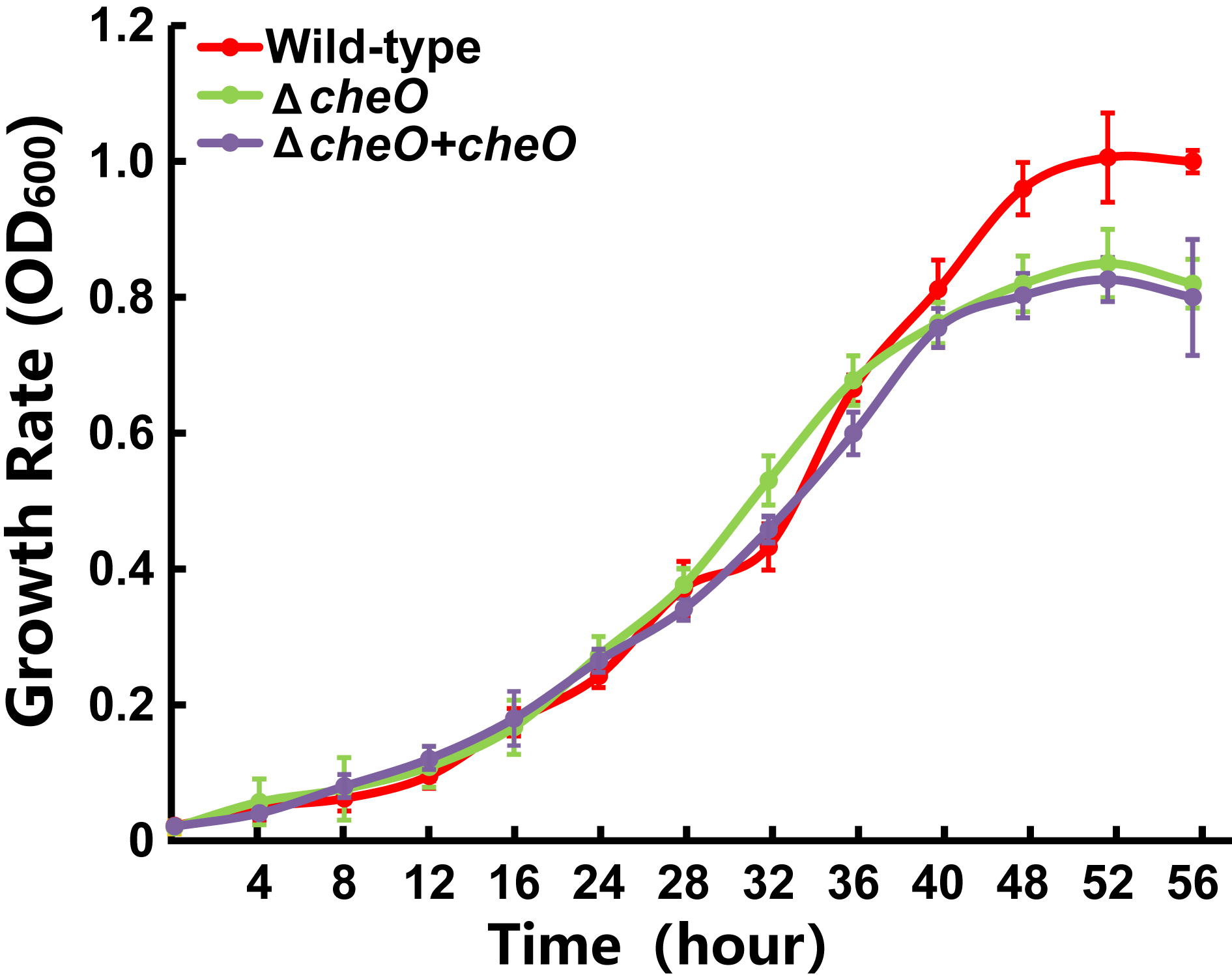

Supplement: S2 Fig — All strains were grown at 37°C in BHI medium in microaerobic conditions. (TIF) [file ppat.1010953.s002.tif]

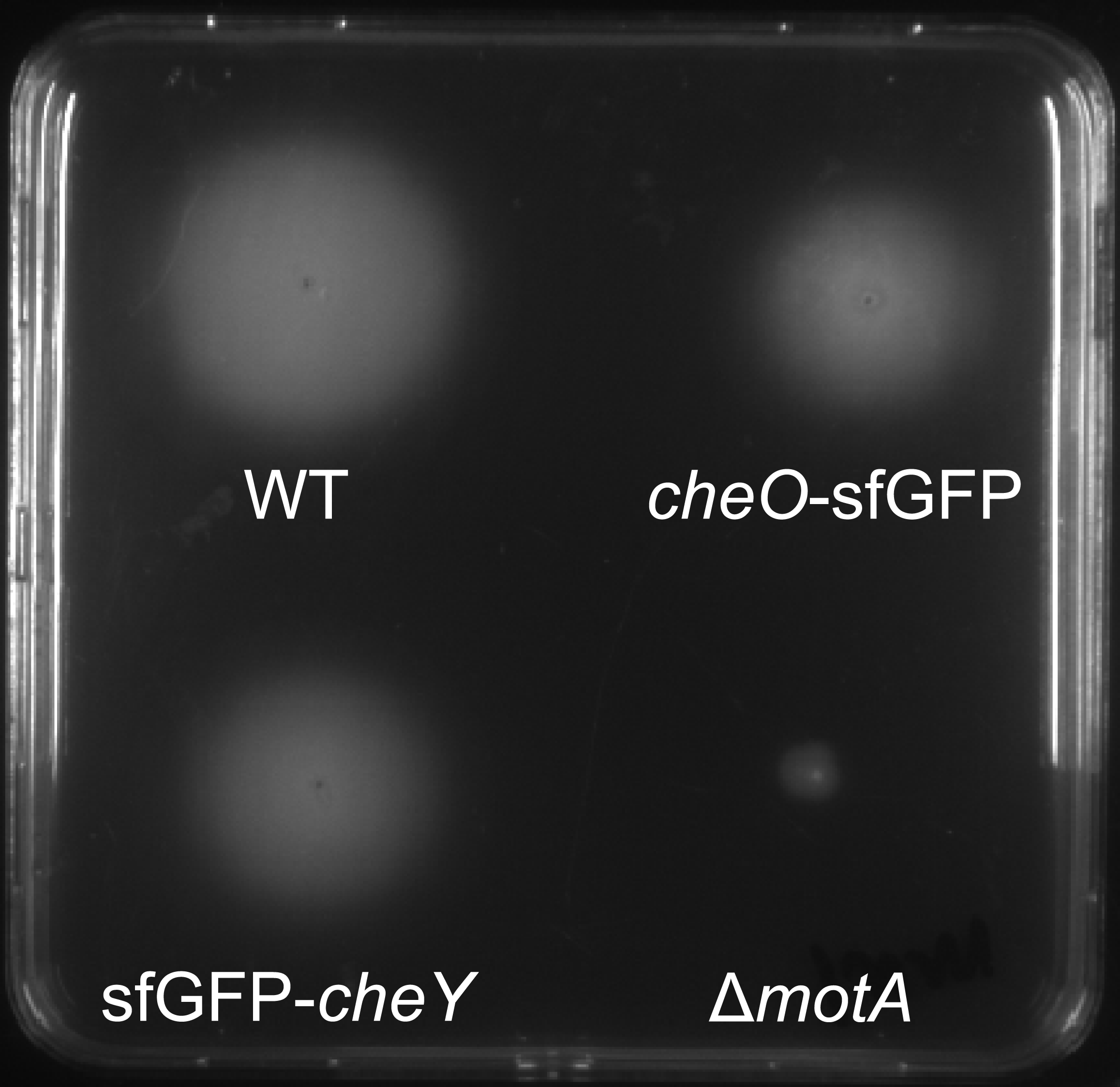

Supplement: S3 Fig — (TIF) [file ppat.1010953.s003.tif]

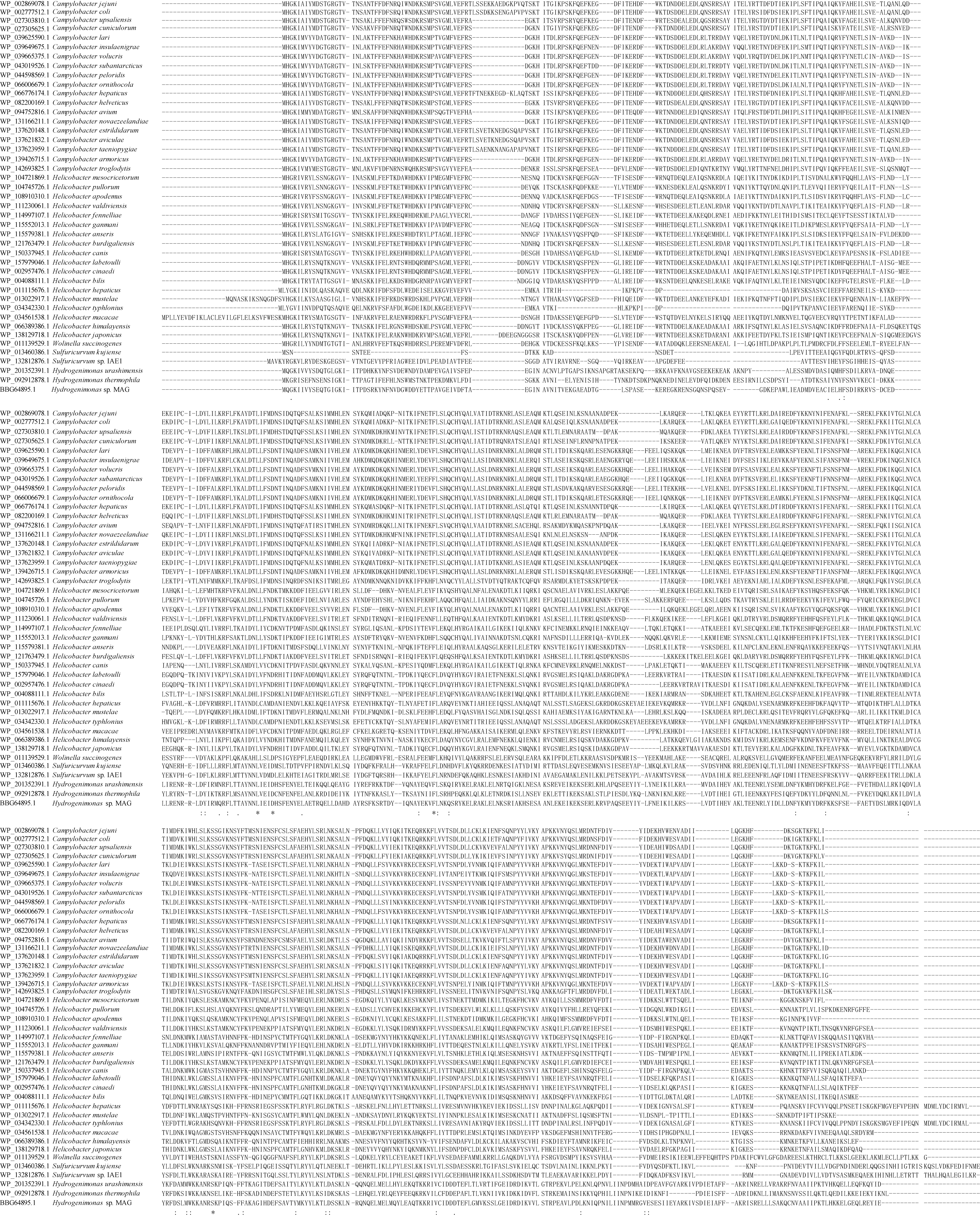

Supplement: S4 Fig — (TIF) [file ppat.1010953.s004.tif]

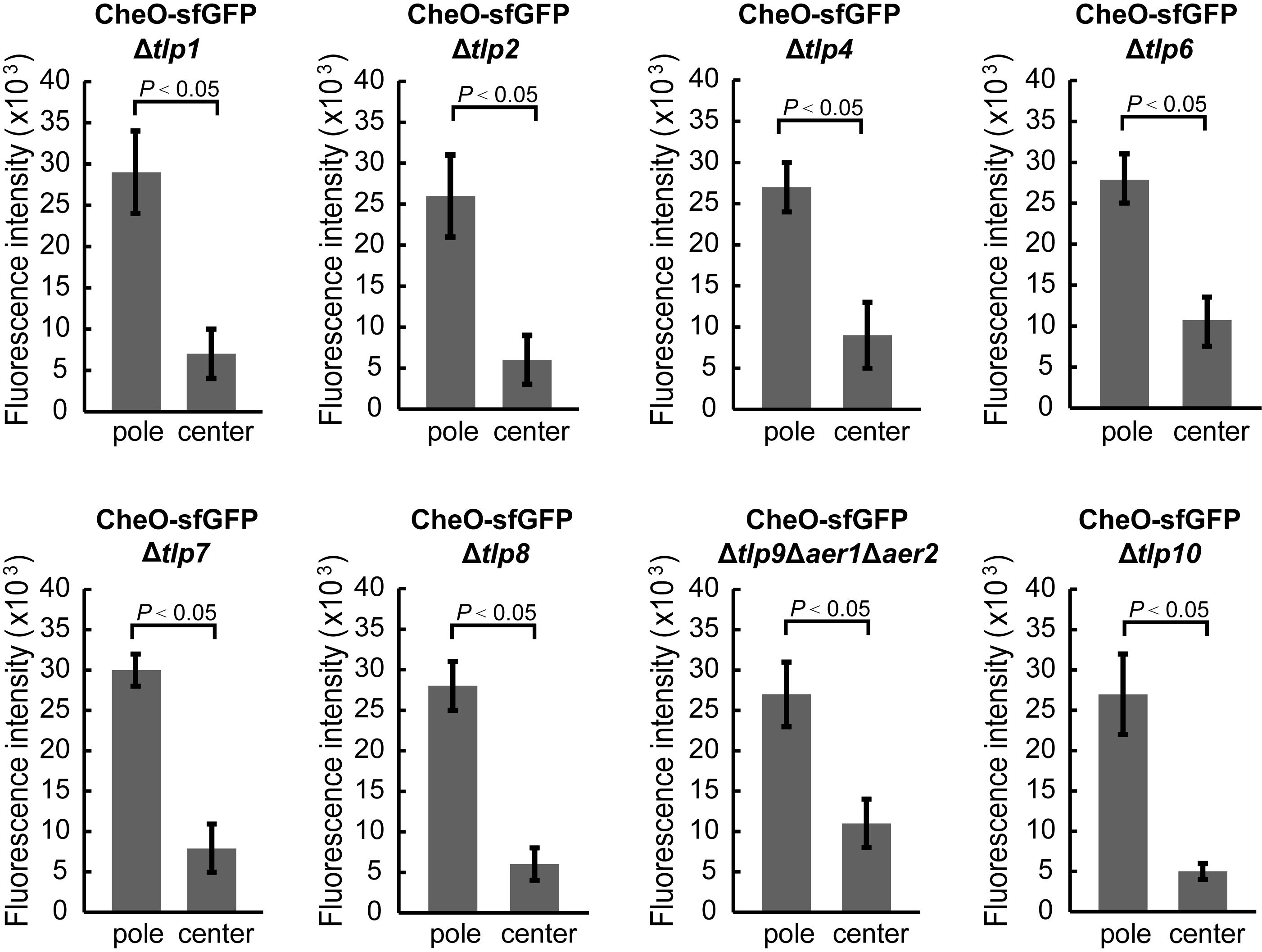

Supplement: S5 Fig — (TIF) [file ppat.1010953.s005.tif]

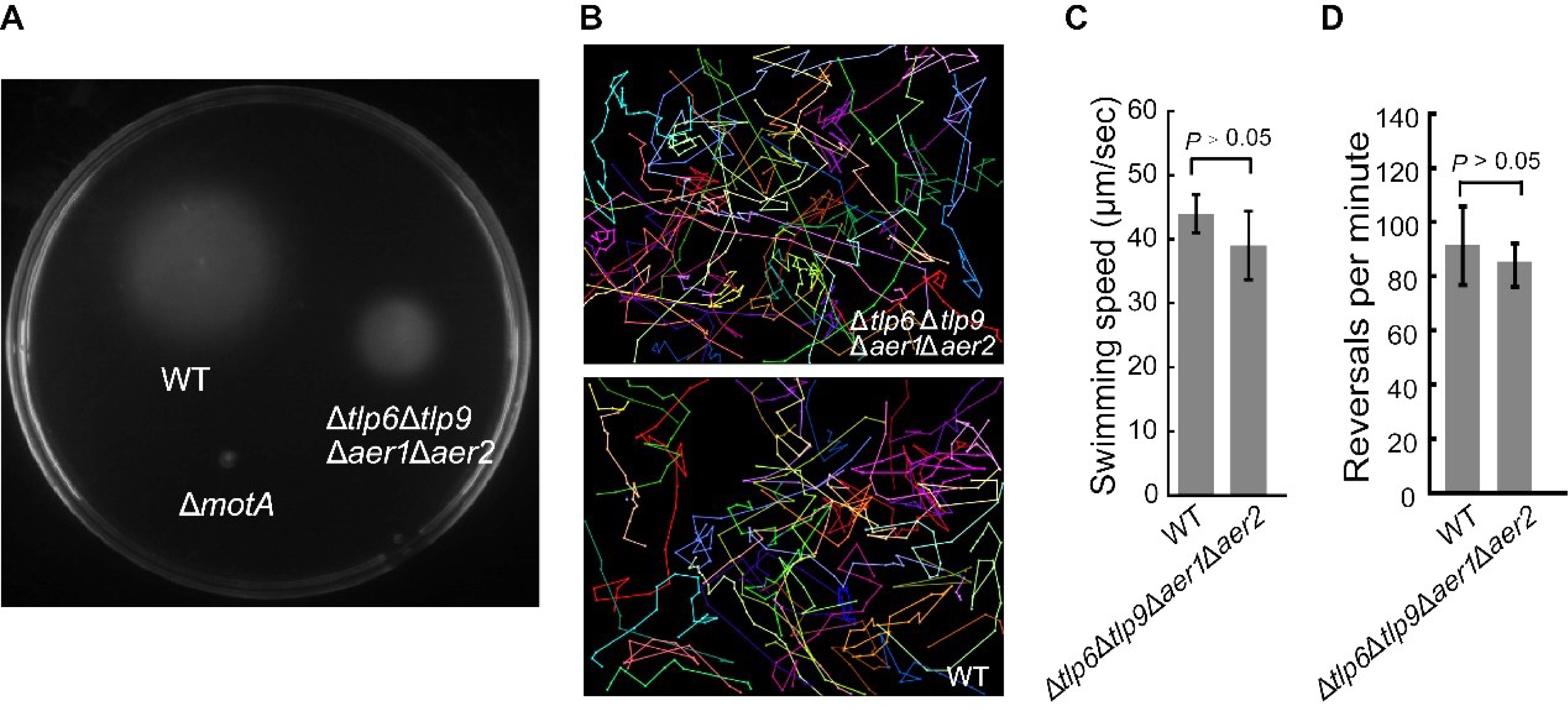

Supplement: S6 Fig — (A) Soft agar motility assay of C. jejuni wild-type, and the Δtlp6Δtlp9Δaer1Δaer2 quadruple receptors mutant strain with ΔmotA mutant as a negative control. (B) Single-cell tracking of C. jejuni wild-type and Δtlp6Δtlp9Δaer1Δaer2 mutant. (C) Quantification of swimming speed of C. jejuni wild-type and Δtlp6Δtlp9Δaer1Δaer2 mutant. Data are shown as mean ± SEM. (D) Quantification of reversal rates of C. jejuni wild-type and Δtlp6Δtlp9Δaer1Δaer2 mutant. Data are shown as mean ± SEM. (TIF) [file ppat.1010953.s006.tif]

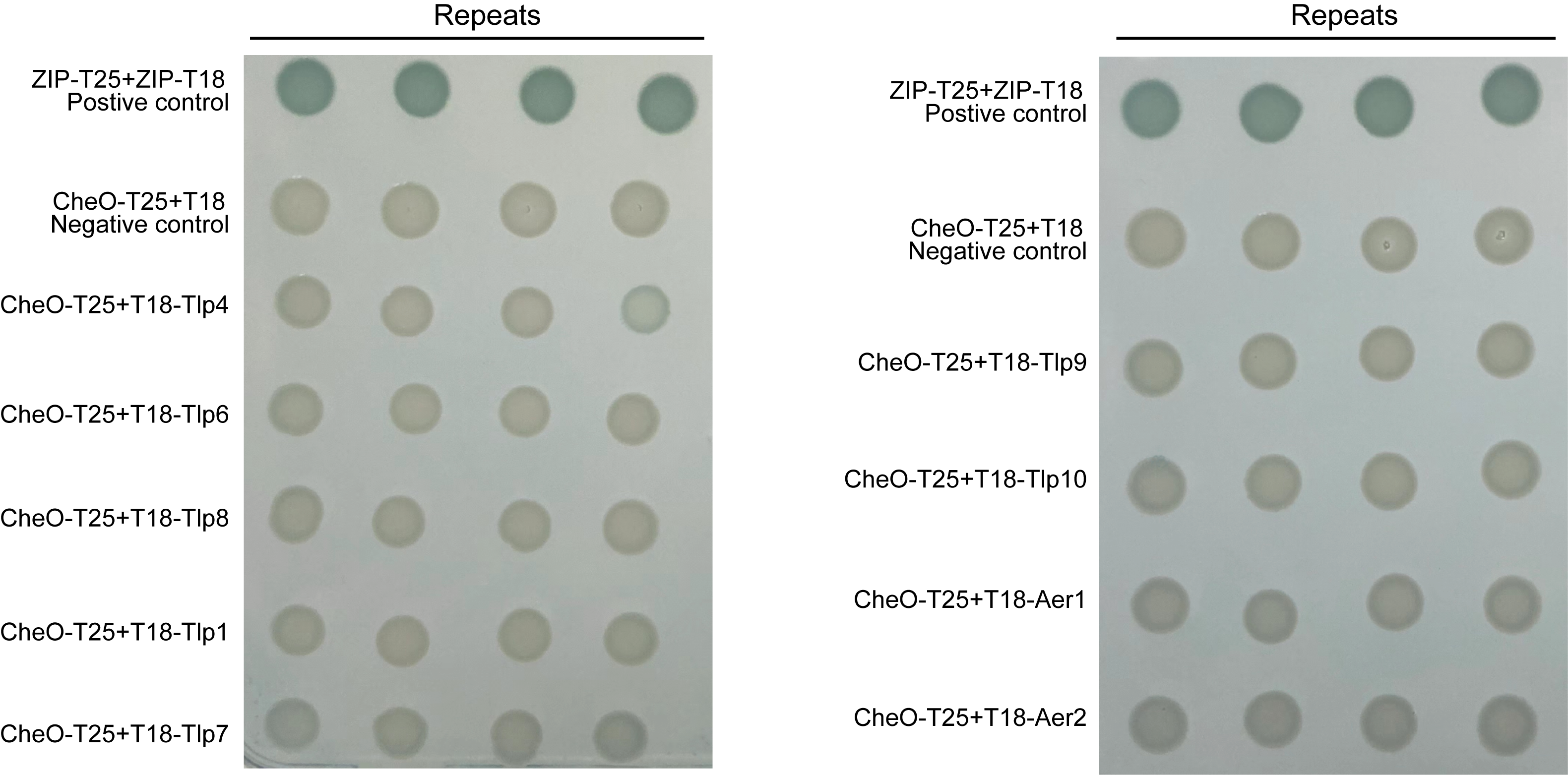

Supplement: S7 Fig — The formation of blue colonies shows that a protein-protein interaction occurs and white colonies show negative results. (TIF) [file ppat.1010953.s007.tif]

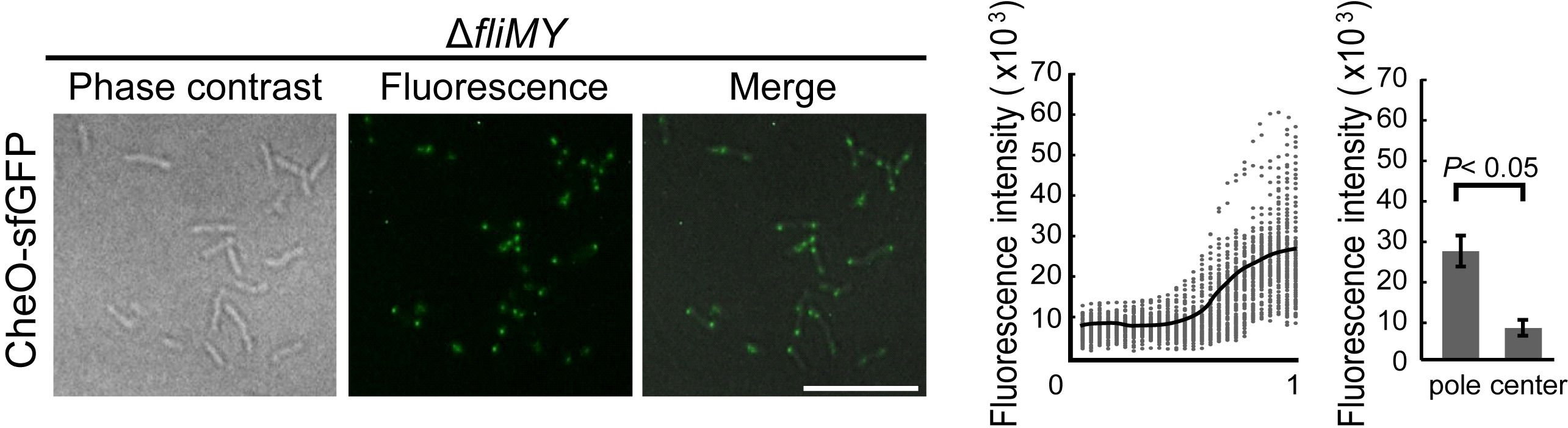

Supplement: S8 Fig — The scatter plot diagram shows the fluorescence intensity of CheO-sfGFP distributed along with the axe of 100 individual cells from the center (0) to the pole (1.0). The black line represents the average intensity of each measuring point. The histogram shows the quantification of the CheO-sfGFP signal intensity at the pole (1.0) and the center (0) of each cell. Data presented as mean ± SEM. Scale bar, 5 μm. (TIF) [file ppat.1010953.s008.tif]

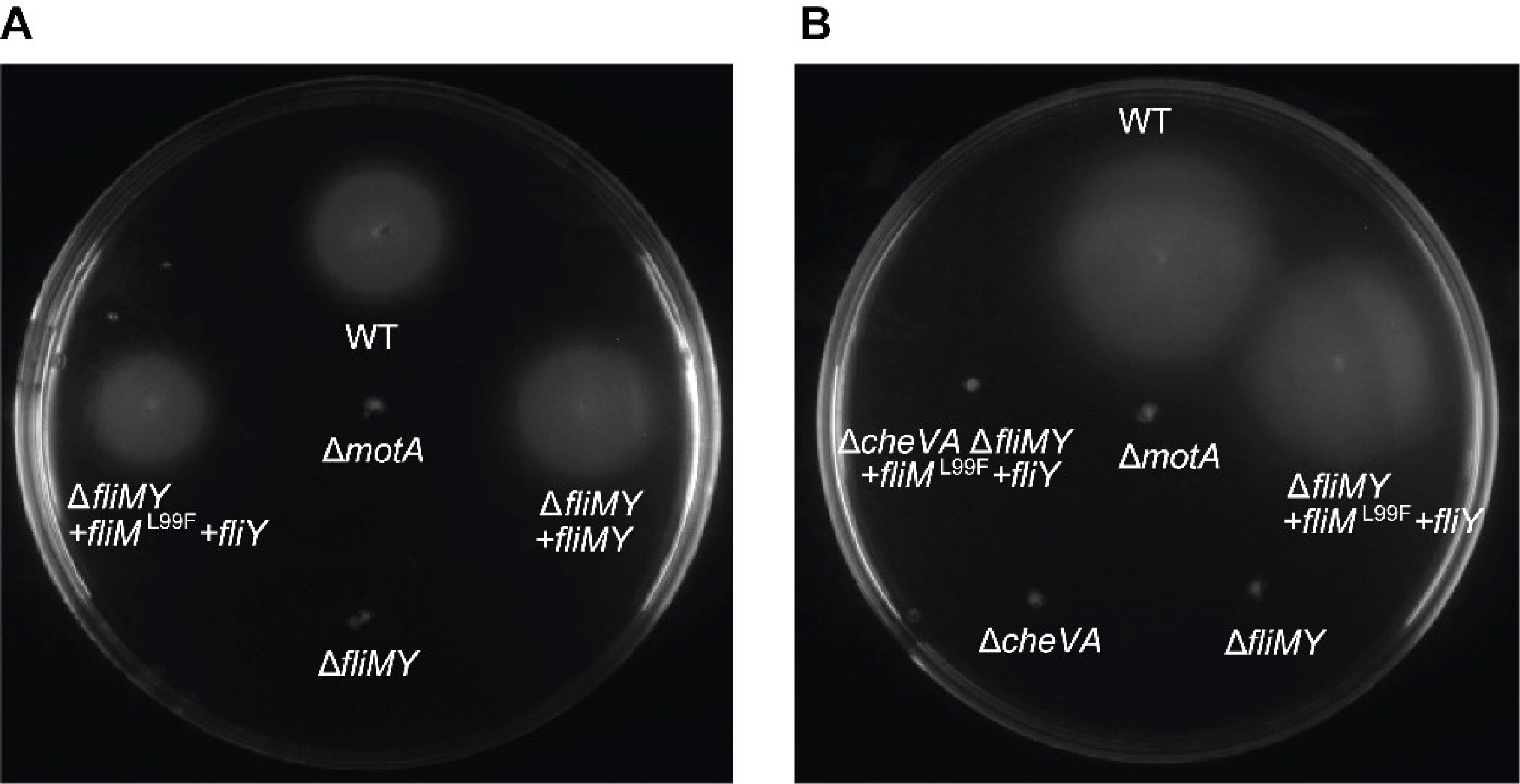

Supplement: S9 Fig — (A) Soft agar motility assay of C. jejuni wild-type; ΔfliMY mutant; ΔfliMY+fliMY strain and ΔfliMY + fliML99FfliY strain. (B) Soft agar motility assay of C. jejuni wild-type; ΔfliMY mutant; ΔcheVA mutant; ΔfliMY + fliML99FfliY strain and ΔcheVAΔfliMY + fliML99FfliY strain. The ΔmotA strain as a negative control. (TIF) [file ppat.1010953.s009.tif]

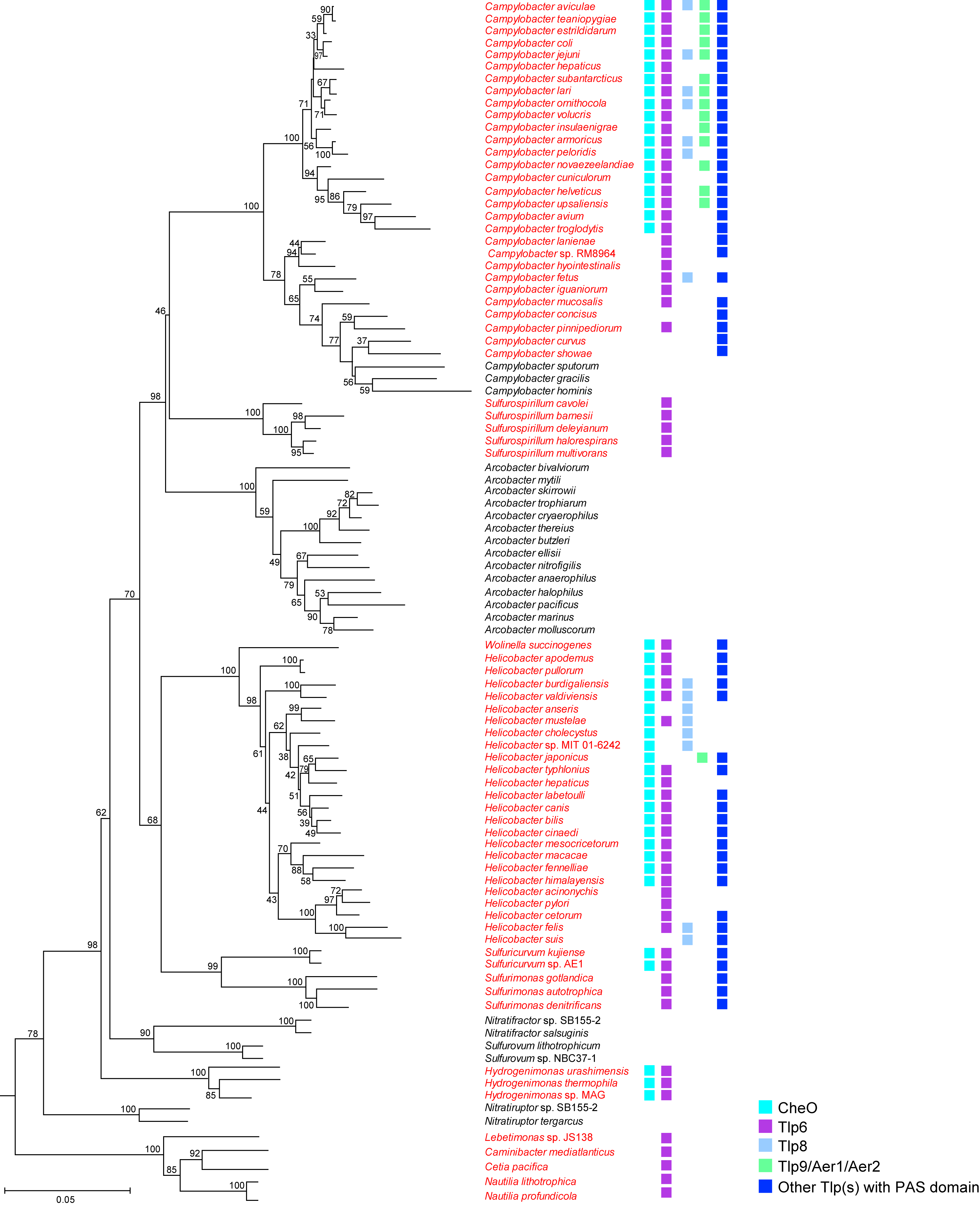

Supplement: S10 Fig — Neighbor-joining phylogenetic tree of Campylobacterota was built from alignments of 16s rRNA. Species containing the F3 chemosensory class are highlighted in red. (TIF) [file ppat.1010953.s010.tif]
