# Supplementary material for: Polar localization of CheO under hypoxia promotes Campylobacter jejuni chemotactic behavior within host
Source: PLoS Pathog. 2022 Nov 3;18(11):e1010953. doi: 10.1371/journal.ppat.1010953 (PMC9665402; doi:10.1371/journal.ppat.1010953)
Supplement: Attachment — Submitted filename: Point to Point reply to reviewers comments.pdf [file ppat.1010953.s014.pdf]

Thank you very much for submitting your manuscript "Polar localization of CheO under hypoxia promotes Campylobacter jejuni chemotactic behavior within host" for consideration at PLOS Pathogens. As with all papers reviewed by the journal, your manuscript was reviewed by members of the editorial board and by several independent reviewers. In light of the reviews (below this email), we would like to invite the resubmission of a significantly-revised version that takes into account the reviewers' comments.

Three experts in the field of bacterial motility and chemotaxis reviewed this work. Overall, the Reviewers concluded that this work is interesting as it identifies a new type of chemotaxis protein in Campylobacter and related species that has the potential to sense or be affected by oxygen tension or redox status. However, two Reviewers believe that there are significant experiments and details lacking in the work that leave too much ambiguity to develop strong conclusions for how CheO functions in chemotaxis in C. jejuni. The work was also reviewed by an editor and the editor largely concurs with all of the Major Issues listed by Reviewers 2 and 3. The authors should seriously consider all of these comments in the Major Issues (along with many of the Minor Issues) to improve the work. Furthermore, an editor notes that significant details are lacking from the Materials and Methods, such as detailed protocol for how the co-immunoprecipitation studies were performed (how were the proteins mixed together - purified proteins, proteins in cell lysates, mixing and washing conditions etc) and other methods that would limit the ability of others to perform similar analyses. The authors should examine this section and provide additional details for this method and potentially others.

We cannot make any decision about publication until we have seen the revised manuscript and your response to the reviewers' comments. Your revised manuscript is also likely to be sent to reviewers for further evaluation.

When you are ready to resubmit, please upload the following:

[1] A letter containing a detailed list of your responses to the review comments and a description of the changes you have made in the manuscript. Please note while forming your response, if your article is accepted, you may have the opportunity to make the peer review history publicly available. The record will include editor decision letters (with reviews) and your responses to reviewer comments. If eligible, we will contact you to opt in or out.

[2] Two versions of the revised manuscript: one with either highlights or tracked changes denoting where the text has been changed; the other a clean version (uploaded as the manuscript file).

Important additional instructions are given below your reviewer comments.

Please prepare and submit your revised manuscript within 60 days. If you anticipate any delay, please let us know the expected resubmission date by replying to this email. Please note that revised manuscripts received after the 60-day due date may require evaluation and peer review similar to newly submitted manuscripts.

Thank you again for your submission. We hope that our editorial process has been constructive so far, and we welcome your feedback at any time. Please don't hesitate to contact us if you have any questions or comments.

Sincerely,

David R Hendrixson
Guest Editor
PLOS Pathogens

Nina Salama
Section Editor
PLOS Pathogens

Kasturi Haldar
Editor-in-Chief
PLOS Pathogens
orcid.org/0000-0001-5065-158X

Michael Malim
Editor-in-Chief
PLOS Pathogens
orcid.org/0000-0002-7699-2064

****

Three experts in the field of bacterial motility and chemotaxis reviewed this work. Overall, the Reviewers concluded that this work is interesting as it identifies a new type of chemotaxis protein in Campylobacter and related species that has the potential to sense or be affected by oxygen tension or redox status. However, two Reviewers believe that there are significant experiments and details lacking in the work that leave too much ambiguity to develop strong conclusions for how CheO functions in chemotaxis in C. jejuni. The work was also reviewed by an editor and the editor largely concurs with all of the Major Criticisms listed by Reviewers 2 and 3. The authors should seriously consider all of these comments to improve the work. Furthermore, an editor notes that significant details are lacking from the Materials and Methods, such as detailed protocol for how the co-immunoprecipitation studies were performed (how were the proteins mixed together - purified proteins, proteins in cell lysates, mixing and washing conditions etc) and other methods that would limit the ability of others to perform similar analyses. The authors should examine this section and provide additional details for this method and potentially others.

Reviewer's Responses to Questions

Part I - Summary
Please use this section to discuss strengths/weaknesses of study, novelty/significance, general

execution and scholarship.

Reviewer #1: This is a very comprehensive, elegant and detailed study of a new chemotaxis regulator in campylobacters. The manuscript is well written, and the study will greatly contribute the chemotaxis field in general. There are a few thoughts for the authors to consider and some minor language corrections:

Response: We thank the reviewer for positive comments.

In your discussion:
Consider postulating that CheO is likely to associate with CheA within the chemosensory array in a manner similar to CheY and that is the reason that once the array formation is destabilised, it does not localise to the poles.

Response: Thanks. We have added this postulation in the discussion, please see Lines 372-375: “Furthermore, CheO is likely to associate with CheA within the chemosensory array in a manner similar to CheY but our *in vitro* phosphorylation assays did not yield conclusive results. Thus, the detailed mechanism of CheO in chemotaxis signal transduction is unclear.”

Line 361-364: Consider that vigorous motility does not really allow for adaptation, but rather allows the cell to sense and relocate to more favourable or preferred oxygen environment/level.

Response: Thanks for the good point. We have changed the sentence as “Moreover, because CheO is more necessary in microaerobic than in aerobic conditions, this protein likely promotes more vigorous chemotactic motility to help *C. jejuni* sense and relocate to niches with preferred oxygen level and other favorable conditions.” (Lines 392-394).

There are a few suggestions for the discussions within the minor comments below:

Reviewer #2: The manuscript describes a chemotaxis protein responsive to oxygen, named CheO. CheO homologs are conserved in Campylobacter, Helicobacter and Wolinella. The authors show that a functional CheOsgfp localizes at cell poles in a CheAVW-dependent manner, suggesting its localization depends on chemotaxis signaling array formation. CheO-sgfp polar localization is observed when cells are grown under microaerobic conditions but is lost under aerobic conditions. The contribution of CheO to chemotaxis is also increased under microarobic conditions compared to aerobic conditions. CheO appears to affect the swimming reversal, but not speed, and the authors provide evidence that it physically interacts with CheA, CheZ, FliM and FliY. The authors also show that CheO polar localization depends on

the presence of energy taxis receptors (tlp6, tlp9aer1 and aer2). The findings are potentially significant and are certainly novel. However, some of the experiments are incomplete and some conclusions are preliminary with the data presented. The discussion is also highly speculative and lacks citation of relevant references. this reviewer suggests a few additional experiments and a more focused discussion and data presentation.

Response: We thank the reviewer for constructive comments. We have performed additional experiments and revised the manuscript accordingly, please see detailed answers in Part II & III.

Reviewer #3: Mo and colleagues report the study of the newly identified CheO protein of C. jejuni. They present a solid characterization showing that cheO is important for chemotaxis particularly under microaerobic conditions. The protein localizes to the pole more under microaerobic conditions, possibly due to interactions with a subset of chemoreceptors. This part of the manuscript is interesting, and could be developed more. The authors show that CheO interacts with multiple chemotaxis and flagella proteins. These findings are well supported, but confusing because it seems surprising for a protein to have such wide interactions, and given that all are present in aerobic and microaerobic, not clear why CheO would lose the interactions and move off the pole. Overall, this work is interesting but would be more powerful if further developed.

Response: We appreciate the reviewer's comments and briefly clarify the confusing part here:

(1) The loss of polar localization of CheO in quadruple mutant Δ*tlp6*Δ*tlp9*Δ*aer1*Δ*aer2* suggests that these energy taxis receptors can affect CheO localization but does not suggest that they directly interact with CheO. We performed new BTH experiment and the results showed that CheO does not directly interact with any chemoreceptors (new Fig. S7). We only confirmed that CheO interacts with CheA, CheZ, FliM and FliY.

(2) New experiment suggest that FliMY does not affect the CheO localization (new Fig. S8);

(3) Δ*tlp6*Δ*tlp9*Δ*aer1*Δ*aer2* retains chemotaxis ability suggesting that the chemosensory array are still formed (new Fig. S6). Based on current results, we suspect that upon sensing oxygen level change, a subset of chemoreceptors can affect the chemosensory array conformation and particularly the CheA kinase, thus indirectly affect the CheO localization and function.

We carefully answered all the questions in Part II & III.

Part II – Major Issues: Key Experiments Required for Acceptance

Please use this section to detail the key new experiments or modifications of existing

experiments that should be absolutely required to validate study conclusions.

Generally, there should be no more than 3 such required experiments or major modifications for a "Major Revision" recommendation. If more than 3 experiments are necessary to validate the study conclusions, then you are encouraged to recommend "Reject".

Reviewer #1: no major issues

Reviewer #2: 1. CheO is proposed to physically interact with CheA, CheZ, FliM and FliY and to localize to the cell poles in a CheAVW dependent manner, as expected as well as in presence of tlp6, tlp9aer1aer2. It is unclear how these multiple interactio0ns as well as an "energy taxis" receptors dependent localization and major role under microaerobic conditions may take place but a few experiments could clarify the findings. Does CheO interact with the energy taxis receptors?

Response: We examined the interaction between CheO and energy taxis receptors Tlp6, Tlp9, Aer1, Aer2 and additional five Tlps (Tlp1/4/7/8/10) by BTH method (**Note:** Tlp 3 and Tlp5 were not included because they are pseudogenes in *C. jejuni* 81-176; Tlp2 was not cloned here because it has an identical cytoplasmic MA domain as Tlp4).

As shown in new Fig. S7 CheO does not interact with any receptors tested here. We added this new result in the Results: "Since the polar localization of CheO is lost in quadruple knockout mutant Δ*tlp9*Δ*aer1*Δ*aer2*Δ*tlp6*, we further examined whether CheO interacts with any of the chemoreceptors through BTH assay. As shown in Fig. S7, CheO does not interact with any of the energy taxis receptor Tlp6, Tlp9, Aer1, Aer2, and the other Tlps (Tlp1/4/7/8/10)" (Lines 286-289).

Does CheO-sGFP localization at the cel poles in aerobic versus microaerobic conditions depends on CheAVW as well as these energy taxis receptors?

Response: Under aerobic condition, CheO-sfGFP lost polar localization in the presence of CheVAW as well as all chemoreceptors as shown in Fig. 3A.

Under microaerobic conditions, Fig. 2A shows that the polar localization of CheO-sfGFP depends on CheVAW; Fig. S5 shows that CheO-sfGFP still localizes at the cell poles in single gene mutants of *tlp1, 2, 4, 6, 7, 8, 10* or in triple gene mutant Δ*tlp9*Δ*aer1*Δ*aer2*; Fig. 3F shows that CheO-sfGFP lost polar localization in quadruple mutant Δ*tlp6*Δ*tlp9*Δ*aer1*Δ*aer2*.

These results suggest that the polar localization of CheO-sfGFP depends on CheVAW and coordinated function of several energy taxis receptors under

microaerobic conditions; the loss of polar localization under aerobic condition is likely related to energy taxis receptors that can affect the chemosensory array conformation and particularly CheA kinase upon sensing oxygen and redox status.

What is the expression patterns of the energy taxis receptors in aerobic versus microaerobic conditions?

Response: To answer this question, we refer to a paper from David J Kelly's group which is a comprehensive study of *C.jejuni* transcriptome and proteome under different oxygen availability (PMID: 28892295). We checked the expression patterns of all chemotaxis genes in this genome-wide study and found that only Tlp6 is significantly up-regulated at both mRNA and protein levels at low oxygen availability, while Tlp9 is down-regulated at mRNA level from high to low oxygen. All chemotaxis genes with differential expression at transcriptional level were summarized in the table below, while their proteomics data are not shown due to no significant changes or low abundance except Tlp6.

Data extracted from Table S1. Microarray data and RT-PCR comparing the 150% and 40% aerobiosis steady states and at time points during the transition from 150% to 40% aerobiosis.

			150% aerobiosis steady-state conditions (1-fold) vs				
C. jejuni 81176 Locus tag	C. jejuni 11168 Locus tag	gene name	5 min	15 min	30 min	60 min	40% Steady State
CJJ81176_0931	Cj0924c	cheB	2.29	1.67	2.33	1.37	2.24
CJJ81176_0311	Cj0285c	cheV	-1.05	-1.68	-1.79	-1.78	-2.47
CJJ81176_0289	Cj0262c	tlp4	-1.63	-3.57	-4.60	-3.61	-1.66
CJJ81176_0473	Cj0448c	tlp6	3.06	6.13	7.07	4.93	1.82
CJJ81176_1205	Cj1190c	tlp9	-2.29	-4.06	-4.50	-4.50	-5.15
CJJ81176_1206	Cj1191c	aer2	-1.91	-2.06	-1.63	-1.33	-1.41

Our results showed that the polar localization of CheO is not altered in Δ*tlp6* or Δ*tlp9*Δ*aer1*Δ*aer2* but is lost in quadruple mutant Δ*tlp6*Δ*tlp9*Δ*aer1*Δ*aer2*, suggesting that the polar localization of CheO depends on concerted effects of more than one Tlps including Tlp6 and Tlp9 (Aer1 and Aer2 are PAS domain alone proteins that interact with Tlp9). But these two Tlps showed distinct expression pattern from high to low oxygen level according to the David J Kelly's study. We understand that the reviewer is interested in how energy taxis receptors can affect CheO localization and function. So far, we can only demonstrate that CheO does not directly interact with Tlps. In addition, the answer below showed the absence of FliMY does not affect CheO localization at the pole. Altogether, we speculate that the energy taxis receptors might affect the chemosensory array conformation to change the interaction between CheA/Z and CheO.

2. The authors provide strong evidence that CheO interacts with FliM (ppi assays, suppressor analysis and role of cheO on swimming reversals). However, the discussion and most conclusions revolve around the link between cheO, microaerobiosis and energy taxis receptors. How does CheO-sgfp localize in mutants lacking FliY, FliM or relative to these

proteins? Are all of these proteins expressed equally in aerobic versus microaerobic conditions?

Response: We introduced *cheO-sfGFP* into Δ*fliMY* mutant and the absence of FliM and FliY does not affect the polar localization of CheO (new Fig. S8). We also added this new result in the Results: "Besides, since the above results suggested CheO directly interacts with two flagellar rotor components FliM and FliY, we investigated whether these flagellar proteins affect CheO localization. A copy of *cheO-sfGFP* was introduced into Δ*fliMY* mutant to replace the native *cheO*, and fluorescence imaging showed bipolar distribution of CheO-sfGFP in *C. jejuni* cells in the absence of FliM and FliY (Fig. S8)." (Lines 289-294).

Both FliM and FliY genes did not show significantly differential expression under different oxygen levels in David J Kelly's study (PMID: 28892295).

3. Discussion : the last two paragraphs lack relevant references that include the published role of Tlp6 CZB in Helicobacter, the subcellular localization of TlpD in H. pylori that is relevant here and the aer1/aer2 and PAS domains of Tlp9 as well as what is known about energy taxis in these different species. Given the multiple interactions of CheO suggested here, additional information on the flagellar motor and chemotaxis in this species should be included. As is, the discussion is speculative and too removed from the existing literature.

Response: The lack of related references in Discussion is an unintentional mistake. We now added all relevant references regarding Tlp6/9 and flagellar rotor and also moderately extended our discussion (see the last three paragraphs in Discussion).

Reviewer #3: Major comments

1. The expression of cheO under aerobic and microaerobic conditions. Fig 3E shows that the expression level of cheO gene remains unchanged in aerobic and microaerobic condition. What about the protein, which could be done by anti-GFP? This is important because if we look at the middle picture of panel A (Fig. 3), the total fluorescence intensity of cheO-sfGFP along the cell body is very different between aerobic and microaerobic conditions. The fluorescence intensity of CheO-sfGFP in microaerobic condition is much higher than that under aerobic condition. The expression level of gfp could affect GFP intracellular distribution.

Response: We performed additional experiment to examine the protein expression level of CheO-sfGFP under both aerobic and microaerobic conditions. For the western blots with anti-GFP antibody and anti-RNA polymerase beta antibody, we controlled the cell lysates and loading volume at the same number for each sample. As shown below, the amount of CheO-sfGFP in equal amount of cell lysates did not show significant differences under microaerobic and aerobic conditions. However, the anti-RNA polymerase beta antibody (for *E. coli*) does not work on *C. jejuni* to serve as a control, so we did not add this result in the manuscript.

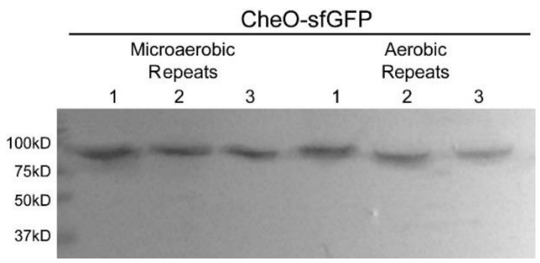

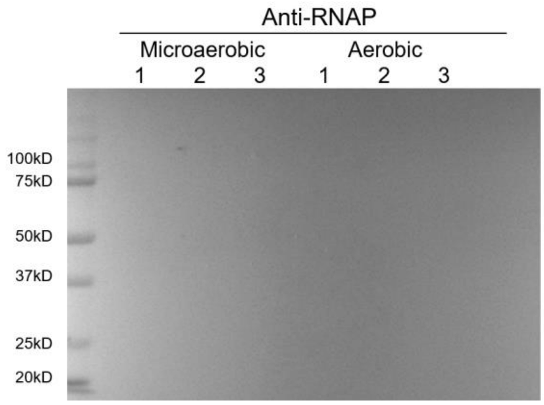

To further clarify this question, CheO is not a transmembrane protein. It is a cytoplasmic protein and the fusion gene copy *cheO-sfGFP* was introduced to *C. jejuni* wild type strain via recombination to replace the native *cheO* gene. So the expression of *cheO-sfGFP* is under native promoter and there are no over-expression of this protein.

2. The result of CheO relocalization is very interesting--but having the GFP fusion could be introducing artifacts. Could authors confirm this the localization of CheO under different conditions using immonofluorescence with untagged CheO, or at least differently tagged CheO?

Response: Thanks for the suggestion. We do not have an antibody for CheO but we have tried immunofluorescence experiments with Anti-FLAG antibody for CheO-3xFLAG fusion protein. The quality of the immunofluorescence images was not as good as the *in situ* CheO-sfGFP expression, and we did not collect enough immunofluorescence pictures for statistics. For the reviewer's concern regarding the CheO-sfGFP fusion, we have two points to clarify:

(1) CheO is a cytoplasmic protein with no transmembrane domain, and the fusion gene copy of *cheO-sfGFP* was introduced into the *C. jejuni* chromosome to replace the native *cheO* copy, thus the expression of *cheO-sfGFP* is under native promoter to avoid over-expression;

(2) We performed soft agar assay with *cheO-sfGFP* mutant, which showed similar level of swarming motility as the wild type (Fig. S3), suggesting that the function of CheO is not altered due to the GFP fusion.

3. The authors show that CheO is dispersed in the quadruple tlp9, aer1, aer2, tlp6 mutant, but not in any single chemoreceptor mutants. This work needs follow up. First, are CheA, CheW, CheV dispersed in these quadruple mutants, e.g. is it specific? Line 241-245 and Fig. S5, not all the single tlp/aer mutants are shown, e.g. no single tlp9, aer1 or aer2 deletion. Also, the mutant labeled by deltatlp9aer1aer2 is a bit unclear-- should it be labeled as deltatlp9 deltaaer1 deltaaer2.

Response: First, we want to clarify that Aer1 and Aer2 in *C. jejuni* are only composed of PAS domain alone, different from the Aer receptor in *E. coli* (Please see Fig. S1 for domain organization of all chemoreceptors in *C. jejuni*). Previous studies suggested that Aer1 (CetB) and Aer2 (CetC) interact with Tlp9 (CetA) to form complex for signal transduction (references 39, 40, 41). Specifically, Tlp9 and Aer1 are co-transcribed and is a bipartite energy taxis system in *C. jejuni* (ref 39, 40). The *aer2* is at the upstream of *tlp9*/*aer1* genes and *aer2* (*cetC*) functionally complements a Δ*aer1* (*cetB*) mutant, required for energy taxis in concert with Aer1 (CetB) (ref 41). Thus, we decided to make a triple Δ*tlp9*Δ*aer1*Δ*aer2* mutant to test whether the complex affects CheO localization. In Δ*tlp9*Δ*aer1*Δ*aer2* mutant, CheO still localizes at the cell poles, so we did not go further to make individual mutant for *tlp9*, *aer1*, and *aer2*. Other than these 3 genes and two pseudogenes (*tlp3* and *tlp5*), we made single gene mutant for all the other *tlps* in *C. jejuni* to test their effect on CheO localization (Please see Fig. S5).

We have renamed Δ*tlp6*Δ*tlp9*/*aer1*/*aer2* as Δ*tlp6*Δ*tlp9*Δ*aer1*Δ*aer2* in the text and Figures.

Our additional experiments described below in answers to question 5 is an indirect evidence to show that CheV, CheA, CheW proteins still form array at the cell poles.

4. It would be great to include double and triple tlp6, tlp9, aer1, aer2 mutants to really understand which receptor(s) are driving the localization.

Response: The answers to the above question #3 clarified why we only generated *Δtlp6, Δtlp9Δaer1Δaer2, and Δtlp6Δtlp9Δaer1Δaer2,* but not more double or triple mutants on these four genes. We understand that the reviewer is interested in how each or more Tlps affect the CheO localization, and we have added an additional experiment in the revised manuscript to check whether CheO interact with any Tlps. As shown in Fig. S7, CheO does not interact with any receptors tested here. We added this new result in the Results: "Since the polar localization of CheO is lost in quadruple knockout mutant Δ*tlp9*Δ*aer1*Δ*aer2*Δ*tlp6*, we further examined whether CheO interacts with any of the chemoreceptors through BTH assay. As shown in Fig. S7, CheO does not interact with any of the energy taxis receptor Tlp6, Tlp9, Aer1, Aer2, and other Tlps (Tlp1/4/7/8/10)" (Lines 286-289).

Based on our current results, more than one Tlps concertedly affect the polar localization of CheO. For future thorough examination on this question, more tlp combinational mutants will be needed, not limited to *tlp6, tlp9, aer1* and *aer2*.

5. What is the soft agar migration of the quadruple receptor mutant?

Response: We performed additional experiments to answer this question, please see the results in new Fig. S6. Basically, the motility ring of Δ*tlp6*Δ*tlp9*Δ*aer1*Δ*aer2* mutant was slightly reduced in the soft agar plate, but its swimming velocity and reversal frequency were not significantly different from the wild-type in the single cell tracking analysis. These results suggested that the quadruple mutant retains chemosensory array structure, i. e. the CheV, CheA, CheW proteins still form array at the cell poles.

6. Were the protein-protein interactions done under aerobic or microaerobic conditions? Do any of them change?

Response: Both protein-protein interaction experiments (BTH and Co-IP) were carried out in *E. coli* strains. The *E. coli* cell were cultured in aerobic condition and we have not examined protein-protein interaction in strictly microaerobic environment.

7. Suppressors, Fig. 5. --The authors should show whether the FliM mutation is specific to cheO, or whether it causes increased migration of other chemotaxis mutants and WT.

Response: We have performed motility assays for Δ*fliMY*, Δ*fliMY+fliMY*, and Δ*fliMY+fliMY*^{L99F} with wild-type strain as a control. As shown in new Fig. S9, *C. jejuni* Δ*fliMY* mutant lost its motility, while complementation of *fliMY* to Δ*fliMY* mutant restored motility. Moreover, the Δ*fliMY+fliM*^{L99F}*fliY* mutant strain showed similar swarming ability compared to the WT and Δ*fliMY+fliMY* strains, i.e. FliM^{L99F} did not enhance the motility of *C. jejuni*.

A copy of *fliM*^{L99F} was also introduced into *C. jejuni* Δ*cheVA* mutant, which did not restore its motility, suggesting that the FliM^{L99F} is not a suppressor of the motility phenotype of Δ*cheVA* mutant.

Part III – Minor Issues: Editorial and Data Presentation Modifications

Please use this section for editorial suggestions as well as relatively minor modifications of existing data that would enhance clarity.

Reviewer #1: Minor corrections:

Line 155: This is not clear, and I think it meant to say that the mutation had reduced, rather

than abolished the ability of bacteria to swarm on the soft agar plate. The mutation did not affect swimming…

Response: We rephrased as "The results showed that mutation of *cheO* had reduced, rather than abolished the ability of *C. jeuni* to swarm on the soft agar plate (Fig. 1B), but did not affect its swimming ability in liquid (Movie S1)." (see Lines 158-160)

Line 157: swarming?

Response: Changed to "swarming".

Line 161, remove coma after serine

Response: Removed.

Line 166, effect ON chemotaxis

Response: Changed to "on".

Line 206, please rephrase the subtitle, it is not clear, perhaps: "Tlps involved in energy…"

Response: Changed to "Tlps involved in energy taxis".

Lines 220-223, please rephrase for clarity.

Response: Rephrased as "In these two experimental setups that were referred to aerobic conditions, CheO-sfGFP did not display bipolar fluorescence loci, in contrast to the circumstance that *C. jejuni* cells were grown and handled completely in a microaerobic workstation (Fig. 3A)".

Line 225, Line 350: swarming rather than spreading?

Response: Changed to "swarming" in both places.

Line 231: not likely to be regulated…

Response: Changed to "not likely to be regulated".

Line 231: residues

Response: Changed to "residues".

Line 237: appears less likely to be able…

Response: Changed to "appears less likely to be able to…..".

Line 246-247: in the following statement, please clarify whether Tlp6 or Aer1/2 are composed (actually: contain) a PAS domain.

Tlp9 interacts with sensor proteins Aer1/Aer2 composed of the PAS domain alone and Tlp6 has a CZB domain

Response: Clarified as "Tlp9 does not contain a PAS domain but it interacts with two sensor proteins Aer1 and Aer2, both of which are composed of the PAS domain alone (Fig. S1) [39, 40]. Tlp6 is a cytoplasmic chemoreceptor with a CZB domain but no PAS domain (Fig. S1) [50, 51]." (see Lines 250-253)

Line148: throughout, rather than "all over"

Response: Changed to "throughout".

Line 249-251: Please consider that it is not the coordinate presence of Tlps that co-ordinately determine that recruitment of CheO, but the mutation you created had destabilised the chemosensory array formation leading to inability of CheO to integrate/associate. I think this is more likely, and in any case should be considered, both here and in discussion.

Response: The same concern was also raised by Reviewer #3 in Major question No. 5. We performed additional experiments to test whether the mutation of *tlp6* and *tlp9aer1aer2* destabilize the array formation, please see the results in new Fig. S6. Basically, the motility ring of Δ*tlp6*Δ*tlp9*Δ*aer1*Δ*aer2* mutant was slightly reduced in the soft agar plate, but its swimming velocity and reversal frequency were not significantly different from the wild-type in the single cell tracking analysis. These results suggested that the quadruple mutant retains chemosensory array structure, i. e. the CheV, CheA, CheW proteins still form array at the cell poles.

Lines 285-296. C. jejuni NCTC 11168GC is a known oxygen adapted variant. It had been previously sequenced and, as compared to the 11168-O, it had a number of mutations, thought to allow it to be oxygen tolerant. Are any of those mutations correlate with your revertant' s point mutations? A nice point to include in your discussion, one way or the other.

Response: Thanks for the suggestion! We have tried to download the complete genome sequence for *C. jejuni* NCTC 11168GC, but it is not available in the NCBI GenBank (https://www.ncbi.nlm.nih.gov/genome/browse/#!/prokaryotes/149/). We cannot find a literature for this strain, either. We found a paper that describes an aero-tolerant *C. jejuni* Bf (PMID: 26594244), but its genome is not available, either.

Line 351: dispersed throughout the cell.

Response: Change to "dispersed throughout the cell".

Reviewer #2: 1. chemoarray is not the term used: it should be chemosensory arrays or chemotaxis signaling arrays.

Response: We replaced "chemoarray" with "chemosensory arrays" throughout the manuscript.

2. lines 264-265: the REC domain of CheA is NOT a CheY but a response regulator domain. it should NOT be labeled as CheAY- this is a hybrid kinase CheA with a C0-terminal REC domain which function has not been established in Campylobacter.

Response: Agree. We have changed to "Particularly, the CheA homolog in *C. jejuni* is a hybrid of kinase CheA with an additional receiver domain at the C-terminus." (see Lines 276-277)

3. lines 271-273: a reference is needed for this statement.

Response: We added references regarding CheA-CheY interaction mechanism here.

4. lines 345-346: the authors note some phosphorylation assays but these are not described in the methods or presented in the data. A role for phosphorylation may also0 not be the most relevant here.

Response: Yes, our *in vitro* phosphorylation results were not shown in the manuscript since we cannot draw solid conclusion based on our results. However, as the reviewer #1 pointed out that we should speculate in the discussion that CheO acts in a CheY-like manner in terms of interaction with CheA. Thus, we modified the sentence as: "Furthermore, CheO is likely to associate with CheA within the chemosensory array in a manner similar to CheY but our *in vitro* phosphorylation assays did not yield conclusive results. Thus, the detailed mechanism of CheO in chemotaxis signal transduction is unclear." (Lines 372-375)

5.line 246: TlpD in H. pylori senses oxidative stress, not oxygen.

Response: Changed to "oxidative stress".

Reviewer #3: Minor comments
8. Abstract--include the cheO gene number to make it easier to compare to other work (CJJ81176_1265)

Response: Added, see line 41: "Here, we discovered that the CheO protein (CJJ81176_1265) is required for *C. jejuni* ……."

9. Please include information about the predicted cheO genomic location, operon, and the 2º structure of the protein. Is it predicted to be cytoplasmic?

Response: Please see lines 152-156: "Sequence analyses of CheO did not identify any known domain or motif that provided a clue for its cellular function. In addition, this protein does not contain any obvious signal peptide or transmembrane region, most likely to be a cytoplasmic protein. In the genome of *C. jejuni* 81-176, *cheO* is a stand-alone gene with 86bp intergenic region to its upstream *guaA* (CJJ81176_1264) and 240bp to its downstream *purD* (CJJ81176_1266)."

10. Line 166-170, single-cell tracking was used to test the role of CheO in chemotaxis (Figs. 1F and G). Is the swimming behavior tracked without adding chemotaxis ligands? The conditions and cognate ligands used in this assay should be indicated.

Response: The swimming behavior was measured in liquid BHI medium without adding additional ligands. We have added detailed information in the Methods.

11. Figure 1C lacks the statistical analysis. The caption about Fig 1F and G is incomplete.

Response: The statistical analysis between the Δ*cheO* mutant and the WT strain was added in Fig. 1C. The caption of Fig. 1F and 1G was modified as "(F) Single-cell tracking of *C. jejuni* wild-type and mutant strains in BHI medium with the microscope slides and coverslips sealed in microaerobic atmosphere. Three individual experiments were performed for each strain and 20-30 cells were tracked in each experiment. The images shown here are representatives for each strain to compare their swimming behavior. (G) Quantification of reversal rates of *C. jejuni* wild-type and mutant strains. The reversal rates were calculated as the number of directional switches per minute per cell and data are shown as mean ± SEM. Differences between the mutant and the wild-type were statistically analyzed by Student's *t*-test".

12. Line 187-189 (Fig. 2A). Is the fluorescence observation done under microaerobic conditions? The description is not clear.

Response: The fluorescence observation was done under microaerobic condition. The sentence were modified as "Fluorescence microscopy showed that CheO-sfGFP localizes exclusively to the poles of *C. jejuni* under microaerobic condition (Fig. 2A)." (Lines 193-194)

13. Figure 3A. The backgrounds of fluorescence pictures is abnormal. Did authors adjust the image contrast or lightness a lot to make the backgrounds vague? Please make this clear in the figure legend and methods.

Response: The microscope slide appeared contaminated with background fluorescence and we replaced a clearer microscope image for Fig. 3A.

14. Figure 3C and D need statistical analysis.

Response: We added statistical analysis in Fig. 3C and 3D.

15. Line 246-247 ‘Tlp9 interacts with sensor proteins Aer1/Aer2…’ needs citation.

Response: references were added, please see line 252 now.

16. Figure 4 shows that CheO interacts with both the HPT and linker region of CheA. Do authors have any idea about how does that work? For this and the other interactions, it might be useful to determine the cognate interacting region of CheO with other proteins/domains?

Response: Thanks for the suggestion. CheO does not contain any identifiable domain or motif to guide reasonable truncation design to study its interaction region. Future structural information may provide details to guide this study.

17. Fig. 4 might work better moved to come before Fig. 3.

Response: Thanks for the suggestion. With the newly added experiments suggested by reviewer #2, we think it is better to remain the order of Fig 3 & 4 and corresponding text.

18. Line 292: The authors should make it more clear that the G297T refers to nucleotides (not AA, as I initially thought).

Response: We have rephrased this sentence as “We replaced the wild-type *fliM* gene with *fliM*^{G297T} (nucleotide mutation) in the chromosome of Δ*cheO* mutant to test whether FliM^{L99F} (the corresponding amino acid mutation) can alleviate the spreading defect in the absence of CheO.”

19. The discussion part is pretty simplified. It looks like that CheO can interact with many proteins including CheA, CheZ, FliM, and FliY. What do the authors believe is the reason that CheO can interact so many proteins with different functions and domains?

Response: The chemosensory system (F3 class) in *C. jejuni* contains several auxiliary components that are not found in the *E. coli* paradigm, such as CheV, ChePep, CheX, ChePQ. In addition, core components also display distinct domain organizations, such as fusion of REC domain in CheA and lack of REC domain in CheB. The identified CheO here is another element that is not found in other model organisms used for chemotaxis studies. We did not identify any known domain or motif in this protein, so it is not a classical component in known chemotaxis signaling pathways. Besides, the CheO homologs in species of *Campylobacterota* are generally not conserved (Fig. S4) and have a higher pI value (~8.5). We have collaborated with a structural lab to crystalize CheO but not successful yet since this protein is not a compact structure in solution. We suspect that the sequence flexibility (low complexity region) might enable more interactions of this protein with the others.

We have moderately expanded our discussion, please see the last three paragraphs in Discussion.

20. Methods: There are multiple plate names that are not clearly defined, e.g. karmali agar, and sometimes use of TSA, Brucella Broth, Blood (not clearly defined). Please check the media used and make sure each is clearly described.

Response: In the revised Methods, we have clarified the media used in each experiment.

21. Figure S3 caption about 'strains expressing cheO-sfGfp or sfGFP-cheY' is unclear, wild-type strain or cognate mutants?

Response: We have modified the Fig. S3 caption as "Soft agar motility assay of *C. jejuni* wild-type, *C. jejuni* strains expressing *cheO*-sfGFP or sfGFP-*cheY* at the native *cheO* or *cheY* loci without other mutations, with Δ*motA* mutant as a negative control."

Figure Files:

While revising your submission, please upload your figure files to the Preflight Analysis and Conversion Engine (PACE) digital diagnostic tool, https://pacev2.apexcovantage.com. PACE helps ensure that figures meet PLOS requirements. To use PACE, you must first register as a user. Then, login and navigate to the UPLOAD tab, where you will find detailed instructions on how to use the tool. If you encounter any issues or have any questions when using PACE, please email us at figures@plos.org.

Data Requirements:

Please note that, as a condition of publication, PLOS' data policy requires that you make available all data used to draw the conclusions outlined in your manuscript. Data must be deposited in an appropriate repository, included within the body of the manuscript, or uploaded as supporting information. This includes all numerical values that were used to generate graphs, histograms etc.. For an example see here on PLOS Biology: http://www.plosbiology.org/article/info%3Adoi%2F10.1371%2Fjournal.pbio.1001908#s5.

Reproducibility:

To enhance the reproducibility of your results, we recommend that you deposit your laboratory protocols in protocols.io, where a protocol can be assigned its own identifier (DOI) such that it can be cited independently in the future. Additionally, PLOS ONE offers an option to publish peer-reviewed clinical study protocols. Read more information on sharing protocols at https://plos.org/protocols?utm_medium=editorial-email&utm_source=authorletters&utm_campaign=protocols
